# Analysis on the Cognitive Impact of Social Mobile Games on Left-Behind Children in the Era of Big Data

**DOI:** 10.3389/fpubh.2022.915801

**Published:** 2022-06-06

**Authors:** Mengyu Li, Jinglei Li, Megat Al Imran Yasin, Mohd Nizam Osman, Norliana Binti Hashim, Lay Hoon Ang, Yitian Xue

**Affiliations:** ^1^School of Journalism and Communication, Zhengzhou University, Zhengzhou, China; ^2^Faculty of Modern Languages and Communication, Universiti Putra Malaysia, Serdang, Malaysia; ^3^Propaganda Department, Hohai University, Nanjing, China

**Keywords:** rural economy, social mobile games, left-behind children, cognitive impact, big data

## Abstract

The popularity of mobile gaming has become a common sight in rural areas, and the problem of left-behind children's mobile gaming has become the biggest challenge faced by rural education, and has become a hot topic in the education sector and society. The stage of left-behind children is the golden period of learning and growth. However, this stage is also the period when they have the greatest fluctuations in their ideology due to various factors such as guardians and youths. With the development of big data, it has been applied to various aspects of people's life. This article is mainly based on qualitative research, with the interview and observation as the main methods, supplemented by a questionnaire survey method. In the empirical analysis part, this article has a certain degree of expansion in data selection and research methods. Compared with previous studies, we increased the scale of the research data, making the research results more meaningful. In the research method, the ordinary least squares method (OLS), the propensity score matching method (PSM) and the two-stage least square method (2SLS) are used, and multiple control variables are selected. The factor analysis of the original scores of the historical knowledge test and the original scores of the two-dimensional cultural value evaluation are carried out to obtain the factor scores of cognitive ability. The emotion of the child is expressed by the depression score, and factor analysis is also performed on the depression score. Cognitive abilities refer to reasoning or thinking, processing speeds, and one's ability to solve problems in novel situations, independent of acquired knowledge. OLS regression results show that left-behind children are inferior to non-left-behind ones in cognitive ability. Moreover, left-behind children are more likely to be emotionally depressed. And whether the children are accompanied by migrant or rural parents, there is no significant difference in their cognitive ability and emotions. Because there are unobservable factors that affect whether children are left-behind and children's cognitive abilities and emotions, the sample may have a self-selection bias. This research focuses on the phenomenon of left-behind children's mobile gaming, revealing the compromised cognitive abilities of these marginalized children groups. Our study might put a wake-up for authorities on the education in rural areas.

## Introduction

In recent years, various mobile games have been surging and swept across the country with strong momentum. As of 2019, the number of mobile Internet users in China was 847 million, an increase of 29.84 million from the end of 2018. The proportion of Internet users using mobile phones to access the Internet has increased by 99.1% from 98.6% at the end of 2018 (1). Among the netizens, the students are the most. Among them, the netizens with junior high school, high school/technical school/technical school degrees accounted for 38.1 and 23.8%, respectively; the netizens with college, university, and undergraduate education accounted for 10.5%, respectively. It can be seen that junior high school students use the Internet more often (2). As of June 2019, the number of national online game users reached 494 million, an increase of 9.72 million from the end of 2018, accounting for 57.8% of the total netizens; the number of mobile online game users reached 468 million, an increase of 8.77 million from the end of 2018, accounting for 55.2% of mobile netizens (3). From this point of view, mobile Internet users will play some mobile games to a certain extent, and mobile games are very popular among junior high school students. The survival of marginalized children's mobile gaming has become a common sight in rural areas, and the problem of marginalized children's mobile gaming has become the biggest challenge faced by rural education, and has become a hot topic in the education sector and society (4). Marginalized children incorporate hand-held games into their lives. Hand-held games have become a way of life and another living space for them. In the process of playing hand-held games, they have generated their own culture and civilization, forming a distinctive two-dimensional cultural landscape (5). However, the outside world's understanding of mobile games is still living under moral “panic.” Therefore, mobile games have raised various criticisms, ignoring their positive significance and inherent cultural value of mobile games. This research cuts into the phenomenon of mobile games of left-behind children in rural areas from the perspective of the two-dimensional culture, which has theoretical penetrating power and profoundness (6).

The mobile game survival phenomenon of left-behind children in rural areas is a product of the times and a generation that has been “sacrificed” under social structural reforms. At the same time, the phenomenon of marginalized children's mobile game survival is the primary issue that rural education needs to face directly, and requires sufficient attention from the education sector (7). For mobile games, we should get rid of all kinds of prejudices and panic, look at new things with a rational and dialectical perspective, attach importance to their derived cultural functions historical values, understand their inner spiritual needs and respect their cultural choices and the right to play, and what needs to be done is to guide rather than prohibit. The evolution of civilized and restrained hand demonstrations of left-behind children in rural areas requires a multi-faceted effort. Family education and school education are the top priorities, and then changes in the social structure, social environment, and ethos are the most important to solve the root of the problem (8). At the same time, there are data showing that the playtime of left-behind children is significantly higher than that of non-left-behind children: “playing 4–5 hours a day” accounted for 18.8 and 8.8%, respectively, and “playing more than 6 hours a day” accounted for 18.8% respectively (9). Therefore, marginalized children have become the main force among young players. The survival of marginalized children in mobile games has aroused the attention of all parties and the appeal of experts, setting off a wave of “turbulent waves.” Under the panic of “playing mobile games is sapping the spirit” the government, experts, society, and distressed parents tried to find various measures, but with little effect (10).

The mobile game survival phenomenon of left-behind children in rural areas has attracted attention from all quarters. However, by searching the literature on mobile games and the two-dimensional culture, it was found that the two overlapped very little. The factor analysis of the historical knowledge level and the two-dimensional cultural value evaluation are carried out to obtain the factor scores of cognitive ability. The emotion of the child is expressed by the depression score, and factor analysis is also performed on the depression score. Therefore, this research analyzes the behavior of the marginalized children's hand march from sociology, and takes an insight into the fetters of the marginalized children and mobile games from the perspective of the two-dimensional culture, explores the deep-level educational sociology of the phenomenon, and at the same time reveals the mobile games. The nature of the phenomenon and the cultural connotation it contains not only provide theoretical results for the research on the phenomenon of left-behind children's mobile games, but also inject blood into the theory of the two-dimensional culture and keep pace with the times. Through the investigation and research on the phenomenon of left-behind children's mobile games, it helps us understand the deep-seated needs and living conditions of left-behind children's mobile game players in the virtual game zone, to better guide the gaming behaviors of left-behind children, and at the same time, to provide a new way out and a new reference for solving the problem of left-behind children's mobile game addiction, and to trigger thinking about rural education and the education of left-behind children.

## Related Work

Some scholars' research on the two-dimensional culture and film is basically focused on aesthetics. They believe that two-dimensional culture changes the aesthetic style of domestic films. This change is manifested in: first, the characterization of characters. The characters are set with “cute” as the element and tend to be soft and beautiful. Second, time and space reconstruction. Using virtual reality (VR) technology to create a virtual space-time, this space-time can travel through different eras, and achieve the ideal utopia for human beings. Third, the method of film narration uses ACG elements. Of course, there are also changes in audiovisual language, values, and plot settings. Wang (11) believes that the two-dimensional culture has an impact on the concept, audience change, and aesthetic aspects of film and television creation. Recent advances in information technologies and data science have enabled convenient access, storage, and analysis of massive on-site measurements, bringing about a new big-data-driven research paradigm (22). With the development of Internet of Things (IoT), 5G, and cloud computing technologies, the amount of data from manufacturing systems has been increasing rapidly (23).

In terms of film aesthetics, Fu and Zhao (12) elaborated on the influence of the two-dimensional culture on the aesthetic tendency of films from three aspects: character image, time and space landscape, and value concept. Next, Li et al. (13) believed that the two-dimensional culture has the effect of reducing the aesthetic cracks in the film aesthetics and enhancing the value of the characters, but at the same time it also has the negative effect of excessive commercial consumption and disintegrating the character archetypes. He believes that the two-dimensional culture has brought unique creation to the film. The method also brings a relaxed, cheerful, vigorous and youthful values that are different from the traditional ones, and establishes a unique aesthetic outlook. Niu et al. (14) inherited the research of predecessors and elaborated on film techniques, film styles and film values, and proposed a kind of aesthetic and healing film aesthetic values. He believed that under the influence of the two-dimensional culture, the narrative method and aesthetics of the film have changed a lot, and the two-dimensional culture is the source of motivation for creating excellent movies to integrate the two and avoid being symbolized and conceptualized as the research focus. Researchers believe that the two-dimensional culture has a negative impact on college students who are self-proclaimed and detached from society. On the basis of questionnaire surveys, they understand their psychological needs, and put forward corresponding countermeasures and opinions from schools, families and themselves (18). Li et al. (15) explained that the “cute” factor in the secondary culture can dispel the “scars” and “cruel” gray tones of traditional youth movies, blend the aesthetics of business, culture, and audiences, and then propose movie narratives which is influenced by the two-dimensional culture. It has a personality and reflects the unique aesthetic style of youth. At the same time, in the pursuit of youth, there is a mentality of escaping from reality and confusion, and a cultural mentality that is both lost and recognized. Regarding the two-dimensional culture to promote the development of movies, Liu and Zhou (16) proposed that according to the characteristics of the two-dimensional culture, it should pay attention to learning from and absorbing its outstanding characteristics, and on this basis, form its own unique style to promote the creation and development of film and television.

In terms of countermeasures to reduce the impact of the two-dimensional culture on students, Yue et al. (17) believes that the two-dimensional virtual culture has unique characteristics, attracting a wide range of young people to join, and also leading to the emergence of primary school students' “Internet addiction”: improving laws and strengthen supervision; parents strengthen education, supervision and care for their children; schools strengthen quality education and enrich after-school life; providing a good growth environment. The research is more specific-self-awareness. It is proposed that the different types of secondary culture and the length of contact time have different effects on the development of self-awareness of junior high school students. The corresponding countermeasures are presented: updating concepts and improving media literacy awareness; secondary media self-discipline, ingenious two-dimensional resources; forming educational synergy to promote media literacy education; giving full play to the main role of junior high school students to improve students' self-awareness level (19, 20). In terms of information dissemination, some scholars have their own unique opinions. They believe that under the influence of the two-dimensional culture, the information they choose is to blur the boundaries of countries, ethnicities and regions, but at the same time they have a strong patriotic sentiment and pay attention to national current affairs. However, they remain skeptical of the official information, and hope that the Internet will answer this. Moreover, they remain calm about the information and do not blindly follow the trend, but they pay more attention to their own interests (21).

## Construction of A Model of the Cognitive Impact of Social Mobile Games on Left-Behind Children

### Cognitive Levels of Left-Behind Children

**Figure 1:**
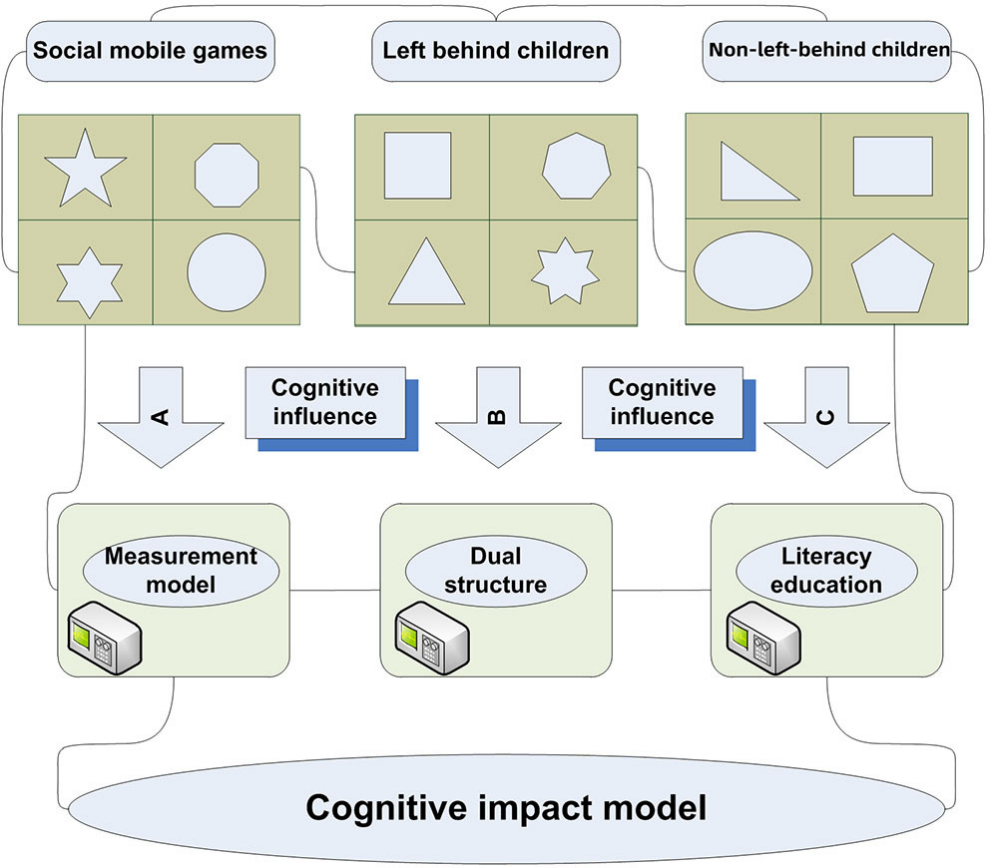
Schematic diagram of the cognitive measurement model of left-behind children.

After several developments, the original connotation is no longer there, but the group of left-behind children has received more and more attention. Left-behind children are the product of the urban-rural dual structure. They are both disadvantaged groups and “marginal people.” Their educational problems and generation methods touch the nerves of society and are difficult to ignore. Figure 1 shows a schematic diagram of the cognitive measurement model for left-behind children.

The first measurement model used in this article is ordinary least square (OLS). Ordinary least squares estimation is to find the estimated values of the parameters, so that the sum of squared deviations of the below formula can be minimized. In the formula, the weight of each square term is the same, which is the parameter estimation method of ordinary least squares regression.


(1) $$\begin{array}{l} X\text{=}\left\{x\left(t\right)\in R|t=1,2,3,..,T\right\} \end{array}$$

The explanatory variable is a definite variable, not a random variable; the random error term has zero mean, same variance, the random error term is not correlated with the explanatory variable; the random error term obeys zero mean, same variance, and zero covariance normally distributed, ordinary least squares estimation is a linear unbiased estimation of the minimum variance of the regression parameters.


(2) $$\begin{array}{l} p\left(X|t\right)=\underset{t}{\sum }p\left(X|z,t\right)p\left(t|x\right) \end{array}$$

Among them, y is the explained variable, including children's cognition and depression (emotion), children is the attention variable, which represents the type of child. Taking the non-left behind children as the benchmark group, we construct left-behind children relative to non-left behind and migrant children relative to each other. As a binaryz duzmmy variable for non-left behind children, *X* is the control variable, including the child's gender, child's age, education investment in the child in the past year, mother's childbearing age, father's childbearing age, mother's years of education, and father's years of education and per capita net income of the household last year, it is a random interference term.


(3) $$\begin{array}{l} p\left(C|X\right)=\overset{T}{\underset{s}{\prod }}p\left(c\left(s\right)|c\left(1\right),c\left(2\right),...,c\left(s-1\right),X\right) \end{array}$$

In order to control the self-selection problem, this paper introduces a propensity score matching model. The basic idea is to find similar individuals in the treatment group (left-behind children/migrated children) in the control group (non-left behind children). The probability of these individuals becoming left-behind children/migrated children is similar and comparable, and can be matched and estimated.


(4) $$\begin{array}{l} t=\ln\;sig\left(\bar{s\left(x\right)}-s\left(\bar{x}\right)\right)=\frac{1}{1+\exp\left(\bar{s\left(x\right)}-s\left(\bar{x}\right)\right)} \end{array}$$

Specifically, the binary treatment variable divides the children in the sample into two groups. For the child individual *i* (*i* = 1, 2, …, *N*), it means that the child is a non-left behind child/migrated child (treatment group), it means that the child is a non-left behind child (control group). There are two potential outcome variables, *x*(0) and *x*(1). As shown in formula, the average processing effect of left-behind children/migrating children interested in this article on cognitive level and emotion is:


(5) $$\begin{array}{l} x\left(t\right)=\{\begin{array}{c} t,0<t<1\\ a*s\left(t\right)+\left(1-a\right)*s\left(t-1\right),t>1 \end{array} \end{array}$$

Among them, *E*[*Y*(1)|*D*(*i*) = 1] is the counterfactual mean value that cannot be obtained from the observable information. Generally, *E*[*Y*(1)|*D*(*i*) = 0] can be used instead, but this method will cause self-selection bias due to factors that affect the type of children and also affect cognitive ability and emotion. The idea of propensity matching score can solve this selective bias.


(6) $$\begin{array}{l} y\left(x\right)-\left(a\left(0\right)+a\left(1\right)*c\left(t\right)+a\left(2\right)*x\left(t\right)\right)=0 \end{array}$$

The propensity matching score needs to meet two conditions. The first hypothesis is the conditional independence hypothesis. The content of this hypothesis is that given a series of control variables, the value of the result variable *Y*(*i*) is independent of the binary processing variable *D*(*i*). The second hypothesis is the common support, that is, the value ranges of individual propensity scores of children with the characteristics of the control variables overlap. If the assumptions are meet, such as the formula, the average processing effect of left-behind children/migrated children on cognitive level and emotion is:


(7) $$\begin{array}{l} A\left(X\right)=E\left[Y\left(1\right)|D\left(i\right)=1\right]-E\left[Y\left(0\right)|D\left(i\right)=1\right] \end{array}$$

Since whether a child is a left-behind child or a migrant child is affected by the choice of parents who go out to work, at the same time, whether the parents go out to work will also affect the child's cognition and emotion, the ordinary least squares method has endogenousness. Table 1 shows the cognitive level distribution table of left-behind children. Therefore, this paper introduces the child type instrumental variable—the proportion of the labor force in the village who go out to work in the total labor force, and uses the two-stage least squares method (2SLS) to solve the endogenous problem of the model.

**Table 1:** Cognitive level distribution table of left-behind children.

**Group index**	**Historical knowledge**	**Two-dimensional cultural value evaluation**	**Overall Cognitive ability score**	**Depressive emotion score**
1	0.261	0.433	0.058	0.228
2	0.441	0.667	0.095	0.366
3	0.221	0.352	0.048	0.178
4	0.132	0.423	0.065	0.281

### Operating Elements of Social Mobile Games

A narrowly defined mobile game refers to a form of the game that can only be run on mobile phones. In a broad sense, mobile games refer to a form of games that can be run on mobile phones and various devices. With mobile phones as the carrier, it is mobile, so it is also called mobile games. With the popularity of mobile phones and the Internet, mobile games have become more popular than ever. There are many types of mobile games and various fancy styles, but Multiplayer Online Battle Arena (MOBA) mobile games are strong and popular among gamers. Among them, “Honor of Kings” is a milestone in MOBA mobile games, pushing MOBA mobile games to a climax. MOBA, also known as a tactical competitive game, is based on strategy and teamwork, emphasizing competition and cooperation. “Honor of Kings” combines role-playing, battlefield kills, intellectual victory, teamwork, and social attributes into one. It not only meets the needs of players for “happy enmity” and a vivid battlefield, but also meets friends and emotions of the need for communication. Table 2 shows the analysis of operating elements of social mobile games.

**Table 2:** Analysis of operating elements of social mobile games.

**Variable item**	**Constant term**	**Sample size**	***R*-squared**
A	1.793	2,355	0.343
B	2.384	2,411	0.289
C	3.012	2,561	0.336
D	2.774	2,713	0.257
E	3.061	2,615	0.281

In people's communication activities, meaningful gestures have begun to work. Significant gestures related to the use of symbols always presuppose that everyone involved in communication activities has the ability to imagine their actions from the standpoint of others and to play the role of another. In symbol communication, people interpret each other's attitudes and actions based on the meaning produced by the interpretation of symbols. The process of human communication involves actors constantly making self-conscious adjustments to the actions of others, that is, repeatedly adapting to each other's actions through definition and redefinition, interpretation and reinterpretation. The application of symbolic interaction theory in this research is embodied in two aspects. On the one hand, the interaction between left-behind children in rural areas and groups in the life world is affected by symbolic interaction theory, including both peer groups and their guardians; on the other hand, the interaction between children and the group in the network virtual space is influenced by the theory of symbolic interaction, and the group mainly includes managers and other game players. When a teammate shows contempt for the equipment of left-behind children in the countryside, they tend to spend more money to buy the equipment. When other players pursue a certain hero, the marginalized children will make the same approach as other game players, and tend to choose the hero.

### Cognitive Impact Model Optimization

**Figure 2:**
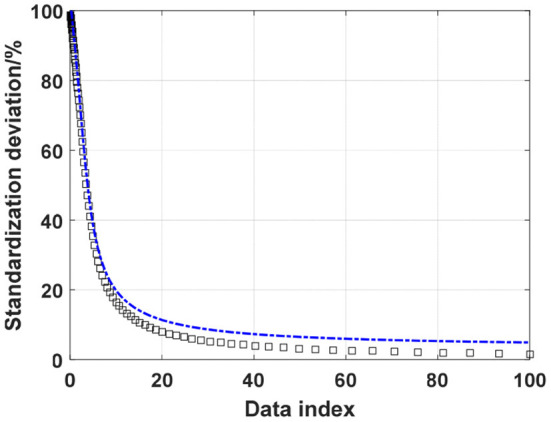
The distribution of the standardized deviation curve of the cognitive impact model.

The mobile game market has become popular recently, which has brought huge economic benefits and also caused a certain negative impact. Among them, marginalized children, as a vulnerable group, are deeply affected when they come into contact with this mobile game. Therefore, this article first defines related concepts such as “left-behind children in rural areas” and “cognition.” The research object is identified as left-behind children in rural areas. Through in-depth interviews with them, we can understand their views on mobile games and their attitudes toward the historical figures in the design. In order to dig out the impact of mobile games on the cognition of left-behind children in rural areas, we further analyze the reasons for this impact, and propose practical and feasible measures to resolve the negative impact. Secondly, we carry out relevant literature collection and draw on the research of related scholars on games and marginalized children. In the model estimation of the left-behind children, the standardized deviations after the control variables were matched were all reduced to <10% except for the quadratic term of the father's age, and the *t*-test results were not significant, that is, the model estimation of the left-behind children satisfies propensity matching to score the assumption that the control variables of the treatment group and the control group are balanced. Figure 2 shows the distribution of the standardized deviation curve of the cognitive impact model. In the model estimation of left-behind children, the standard deviation of most control variables after matching fell below 10%. Although the per capita net income of the family is above 10%, it has dropped a lot from the 24.3% before matching. We satisfy the assumption that the control variables of the propensity matching score treatment group and the control group are balanced.

This article mainly adopts qualitative research methods, including the interview method, and observation method. We read a large number of literature materials, based on previous studies, through induction, comparison, analysis and other steps, to understand the current academic research on the impact of mobile games on marginalized children's cognition abilities, and put forward our research direction, and refer to relevant research results in a targeted manner. It is a face-to-face purposeful conversation between the researcher and the researchee. According to whether the interview is structured or not, it is divided into structured interviews, semi-structured interviews and unstructured interviews. This study mainly adopted a semi-structured interview method, and mainly selected six marginalized children as interview subjects. During the survey process, as participants, we carefully observe the emotional expression of marginalized children during interviews, and observe the family environment of the left-behind children in rural areas to understand the family's educational atmosphere, and observe their verbal expressions when answering questions to prove the authenticity of the answers.

## Application and Analysis of the Model of Social Mobile Games' Cognitive Impact on Left-Behind Children

### Cognitive Analysis of Left-Behind Children in Rural Areas

**Figure 3:**
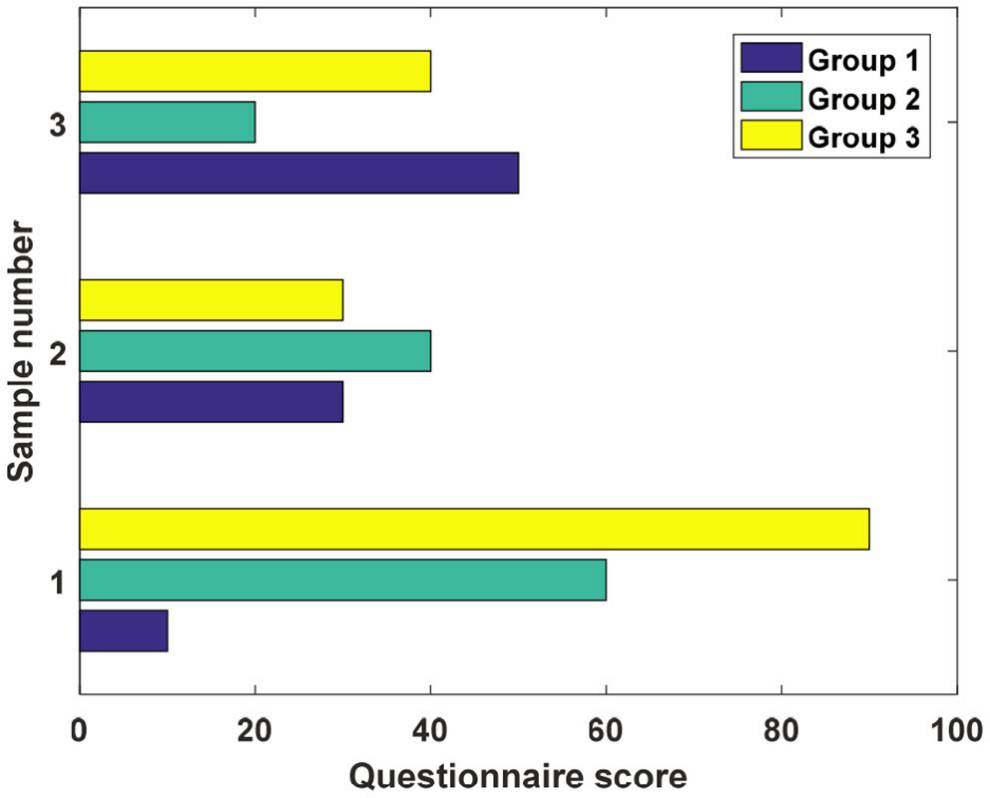
Histogram of cognitive ability scores of left-behind children in rural areas.

According to the research objectives, this article mainly focuses on the development of questionnaires for left-behind children in rural areas. Distributed 466 questionnaires for students, 349 for left-behind children, 117 for non-left-behind children, excluding 8 doodles and incomplete questionnaires, and 115 valid questionnaires for non-left behind children were recovered. The recovery rate was 98.2%. There were 343 left-behind children, and the recovery rate was 98.3%. It also conducts a scientific and systematic analysis of the statistical data, provides a factual basis for the education status of left-behind children in rural areas in the region, and provides microdata support for the study. This article mainly compiles the questionnaire from the basic structure of the family of left-behind children and non-left behind children, the strength of family nurturing functions, and the children's performance in historical knowledge level and two-dimentional cultural value evaluation. The basic structure of the child's family includes who the guardian is at home, the education level of the guardian. Whether it is an only child, the parenting function of the family includes the care and supervision of guardians and parents of migrant workers in their children's historical knowledge level and two-dimensional cultural value evaluation, which includes the level of knowledge about historical figures and stories (such as narrative, ancient poetry, extracurricular reading, etc.), as well as attitudes and evaluations related to two-dimensional culture. Figure 3 shows the histogram of the cognitive assessment scores of left-behind children in rural areas.

It can be seen that about 86.1% of left-behind children are taken care of by their grandparents (grandparents or grandparents), about 8.7% of left-behind children are taken care of by uncles and uncles (or relatives), and left-behind children living alone account for 5.2% of the total number of left-behind children. After controlling for the endogenity of the model, the type of child has no substantial effect the performance in historical knowledge level. The significant effect in the OLS model is due to the inclusion of other uncontrolled unobservable factors. The gender of children has no significant effect on their original scores on the performance in historical knowledge level, but for every year the children's age increases, their original scores on historical knowledge test increase by 1.447 on average, and this result is significant at the 1% level. Table 3 shows the standardized deviation distribution of the control variables before and after model matching.

**Table 3:** Standardized deviation distribution of control variables before and after model matching.

**Attributes number**	**Group A**		**Group B**	
	**Before matching**	**After matching**	**Before matching**	**After matching**
Age	9.5	5.8	2.4	1.7
Gender	4.8	7.2	5.9	6.7
Educational investment	12.2	11.2	2.9	3.6
Net income per capita	5.6	7.4	11.6	9.2

There is no significant impact on children's educational investment, mother's childbearing age, mother's childbearing age square item, father's childbearing age square item, mother's years of education, and children's performance in historical knowledge level test raw scores. The original score on the historical knowledge test was reduced by 0.275, and this result was significant at the 10% level. For every additional year of the father's education years, the original score on the children's historical knowledge level test increases by an average of 0.195, and this result is significant at the 1% significance level. Household net income per capita has no significant effect on the raw scores of children's historical knowledge test.

### Evaluation of Social Mobile Games in the Rural Economy

Because of regional differences and informants are affected by some objective conditions, the subjects of the interview program are only students in 11 classes from the second to sixth grade of elementary school, of which there are two classes in each of the second, third, fourth, and fifth grades, 51 informants were obtained by purposeful sampling. The number of each class are: 44 in class 2 (1) and 11 left-behind children; 47 in class 2 (2) and 12 left-behind children; 41 in class 3 (1) and 8 left-behind children; class 4 (1) with 46 people, 10 left-behind children; class 4 (2) with 45 people, 14 left-behind children. There are 42 students in the class and 12 left-behind children. There are three classes in grade six, each with 40 students in class six (1) and nine left-behind children; 39 students in class six (2) and 10 left-behind children; 40 students in class six (3) and 13 left-behind children.

**Figure 4:**
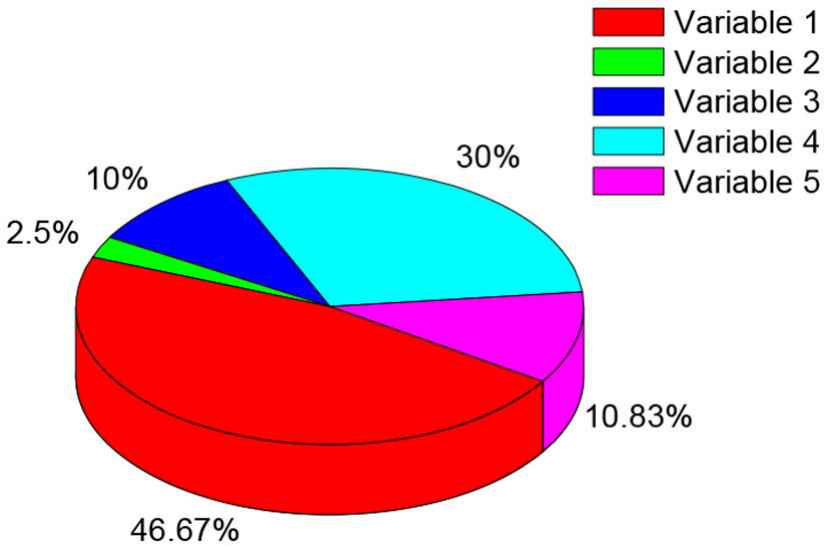
Cognitive abilities distribution of left-behind children among the explained variables.

Figure 4 shows the cognitive fan distribution of left-behind children among the explained variables. The child's cognition in the explained variable uses a set of two-dimensional culture value questions and a set of historical questions to test and evaluate the cognitive level of all respondents who need to complete the self-answer questionnaire (that is, children aged from 10–15 years old). The factor analysis of the original scores of the historical test and the original scores of the two-dimensional cultural evaluation test is carried out to obtain the factor scores of cognitive ability. This article's measurement of children's emotions (depression) combines six related questions in the questionnaire: “How often did you feel depressed, and unable to do anything when you played “Honor of Kings”? How often did you feel nervous when you played “Honor of Kings”? How often did you feel restless and difficult to stay calm when you played “Honor of Kings”? How often did you feel hopeless in the future when you played “Honor of Kings”? How often did you find it difficult to do anything when you played “Honor of Kings”? When you played “Honor of Kings”, do you think life is meaningless?” According to their own circumstances, the interviewees chose the answer that best suits them among the answers: “never, sometimes, half the time, often, almost every time.” For answering option: “never = 1 point; sometimes = 2 points; half the time = 3 points; often = 4 points; almost every time = 5 points”. This article uses two measurement methods. The first is to add the answers to the six questions to the depression score, and the second is to perform factor analysis on the answers to the six questions to obtain the factor score of depression.

### Example Application and Analysis

**Figure 5:**
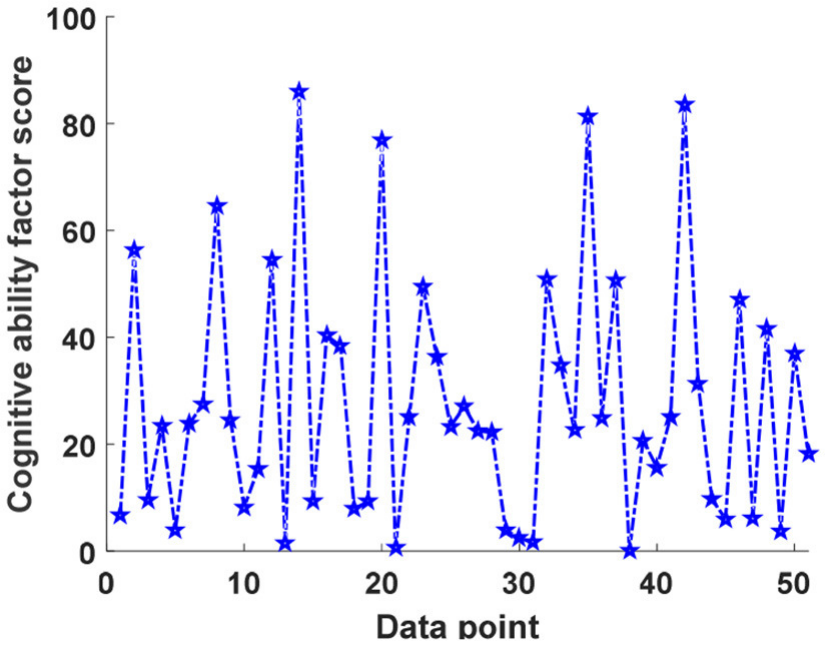
Line chart of cognitive ability factor scores and depression factor scores for different data points. The *symbol indicates the child who takes the cognitive ability test. Y-axis indicates score. There are 51 children in the test.

It can be seen that the average original score of the historical knowledge test for the full sample is 10.51 points, that is, 10.51 of the 24 questions for non-left children with registered permanent residence are answered correctly, but the standard deviation is 4.53, and the fluctuation is large. A total of 34 test questions were designed for the two-dimensional cultural value evaluation. On average, 20.81 questions were answered correctly by non-left children, but the standard deviation was 7.51, and the scores between children were quite different. The children's total scores for the 6 emotional questions answered were 26.99 points. The overall children interviewed had a more positive emotional state. Figure 5 shows a line graph of cognitive ability factor and scores depression factor scores for different data points. Cognitive ability factor scores and depression factor scores are derived from factor analysis, and the mean value is 0, and the variance is both 1.

Among the interviewed children, non-left children, left-behind children, and migrant children accounted for 31, 49, and 20%, respectively. The proportion of non-left children was very high, and the proportion of left-behind children was the lowest. Among the interviewed children, boys accounted for 53% and girls accounted for 47%. There were slightly more boys than girls, all aged from 10–15 years old. The average education investment per child is 1,600 yuan per year, and the average education investment is at a relatively low level, and the standard deviation is 2.65, which is quite fluctuating. The average childbearing age of the interviewed mother is 26.34 years old, and the father is 28.03 years old. The average childbearing age of fathers is slightly higher than that of mothers. The average childbearing age is within the normal range, but the minimum childbearing age of the interviewed mothers is 20 years old and the youngest father is 22 years old. Similarly, some villagers do not have a scientific understanding of the issue of childbearing age. The mother's average education years is 8.22 years, and the father's 9.82 years. The parents' educational level is relatively low, and the grandparents' education years are at least 1 year, which is basically equivalent to ignorance. This will definitely have a negative impact on the children's cognition. Parents with low educational level generally get married and have children earlier. The average per capita net income of households is 6,350 yuan, which is much lower than the urban per capita income level, indicating that the national urban-rural income gap is still not optimistic. The standard deviation is 5.97, indicating that the per capita net income of the interviewed households is also very different.

**Figure 6:**
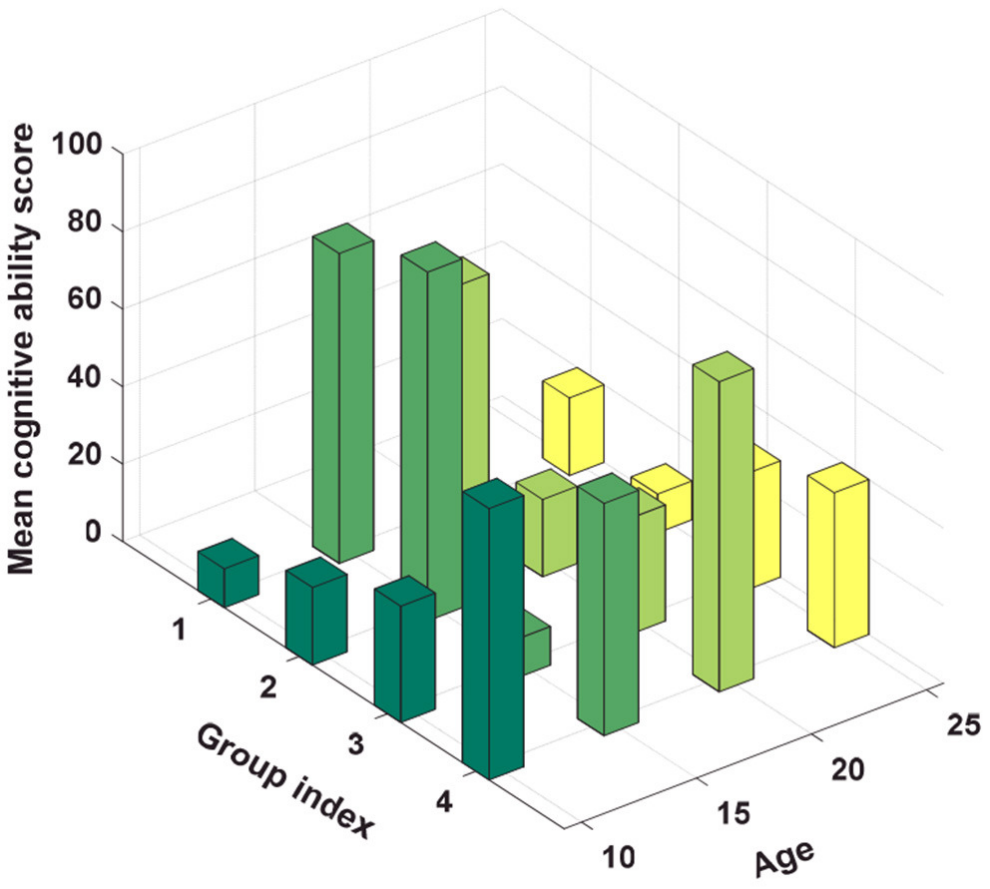
A three-dimensional histogram of the average cognitive ability scores and depression factor scores of left-behind children in different age groups.

The average proportion of the labor force working outside the village is 33.48%. There is a large gap in the proportion of the labor force going out in different regions. This is directly related to the imbalance in national economic development. It is also mentioned in the analysis of the current situation of children that the economically backward central and western regions have a relatively high proportion of migrant workers, while the economically developed eastern regions are much lower. Left-behind children are also mainly concentrated in the central and western regions, and in the economically developed regions. Figure 6 shows a three-dimensional histogram of the average cognitive ability scores of left-behind children in different age groups. The average depression scores of non-left behind children, left-behind children, and migrant children were 77.11, 86.37, and 66.96, respectively. From descriptive statistics, it can be seen that non-left behind children rarely have depression, followed by children with migration. Left-behind children are most likely to have depression emotions. The depression score is the direct sum of the interviewed children's scores for answering six questions, and the depression factor score is a factor analysis, which is equivalent to standardizing the interviewed children's depression scores, which can be seen from descriptive statistics. The scores of depression factors of left behind children are the highest, and non-left behind children are the lowest.

**Figure 7:**
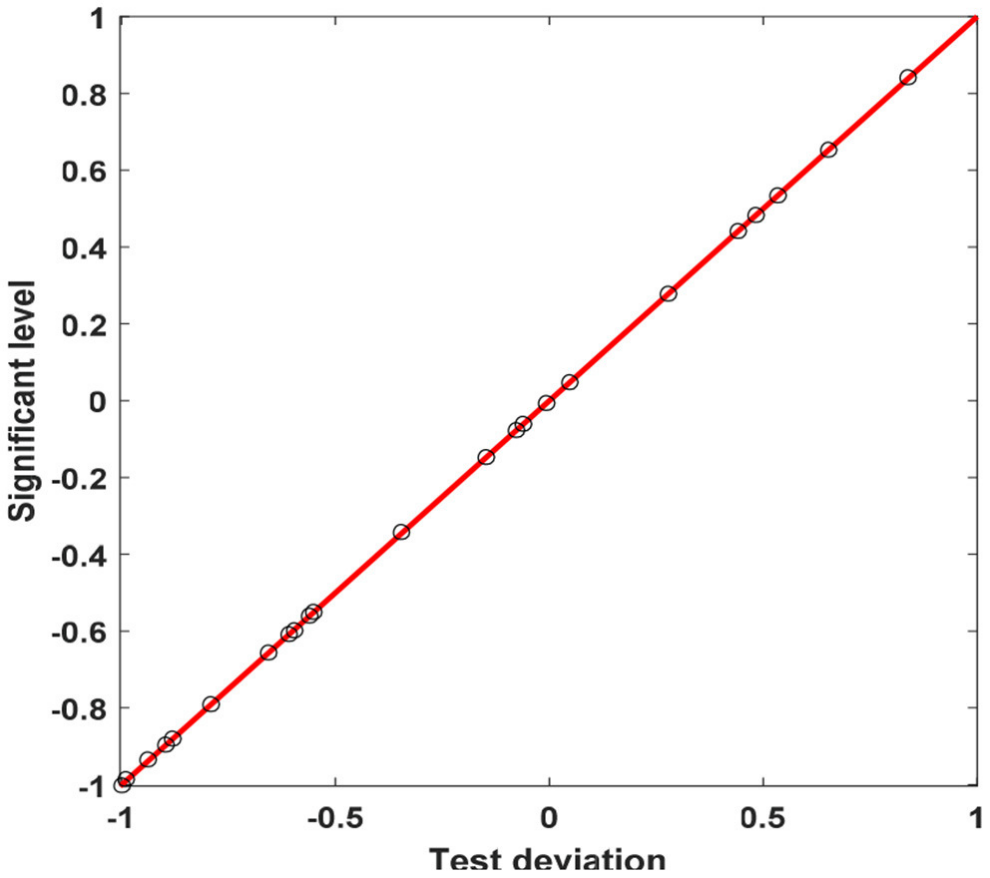
Significance level distribution of cognitive ability test of left-behind children.

Figure 7 shows the distribution of the significance level of the cognitive ability test for left-behind children. On the premise that other conditions remain unchanged, the average original score of left-behind children in the two-dimensional cultural value test is 0.585 lower than that of non-left children, but this result is not statistically significant. On average, the original scores of male children's two-dimensional cultural value evaluation tests are 1.308 lower than female children, and this result is significant at the 5% level. However, gender has no significant effect on the original scores of two-dimensional cultural value test. Every time the child's age increases by 1 year, the original score of the two-dimensional cultural value test will increase by 1.435 on average, and this result is significant at the 1% significance level. For every additional 1,000 yuan invested in children's education, their original two-dimensional cultural evaluation scores increase by 0.195 on average, and this result is significant at the 5% level of significance. For every additional year of mother's years of education, the original score of the children's two-dimensional cultural value test increases by an average of 0.177, and this result is significant at the 5% level. For every year of father's education, the average increase of the original score of the children's two-dimensional cultural value test is 0.458, and this result is significant at the 1% significance level, because fathers generally pay more attention to children's learning. For every 1,000 yuan increase in family net income per capita, the original score of children's two-dimensional cultural value test increases by 0.032 on average, but this result is not statistically significant.

## Conclusion

The stage of left-behind children is the golden period of learning and growth. However, this stage is also the period when they have the greatest fluctuations in their ideology due to various factors such as guardians and youths. The popularity of mobile gaming has become a common sight in rural areas, and the problem of marginalized children's mobile gaming has become the biggest challenge faced by rural education, and has become a hot topic in the education sector and society. This research focuses on the impact of mobile games on the cognition of left-behind children in rural areas, the reasons for the impact, and suggestions on how to solve the negative impact. Through research, it is found that mobile games have two main influences on the cognition of left-behind children in rural areas: first, the positive impact is conducive to expanding the knowledge reserves of historical figures and two-dimensional culture of left-behind children in rural areas, and is conducive to promoting these marginalized groups in learning and interpreting those knowledge. Secondly, the negative impact is in the microfocus, dispelling the right image of traditional historical or two-dimensional culture figures, is not conducive to the formation of a clear knowledge construction; in the macro structure, it is not conducive to the formation of a correct view of history or two-dimensional values for left-behind children in rural areas. Because unobservable factors affect the types of children and a series of dependent variables in this article, this paper selects instrumental variables to conduct empirical analysis again. However, there are still limitations in this study. This experiment uses the achievements related to historical knowledge level and two-dimensional culture evaluation of left-behind children as a reference, but the reliability as a single factor needs to be studied. In the future, multiple factors should be used for analysis at the same time. The results of instrumental variable regression of all groups indicate that whether staying or not staying in rural area, children accompanied by parents have no significant difference on their cognitive abilities and emotions. And furthermore, the left-behind children suffered significant impact on their cognitive abilities and emotions, compared with the non-left behind ones. This article adopts the method of horizontal comparison and vertical comparison. Horizontal comparison is to compare the various learning behaviors and academic performance of left-behind children and non-left-behind children in the process of cognitive abilities. Longitudinal comparison is to compare left-behind children within themselves, so as to find out the differences among left-behind children among the problems of left-behind children, so as to adopt different suggestions and policies for different left-behind children when making suggestions and countermeasures.

## Data Availability Statement

The original contributions presented in the study are included in the article/supplementary material, further inquiries can be directed to the corresponding author.

## Ethics Statement

This research involving human participants is reviewed and approved by the Professor/Academic Committee of School of Journalism and Communication, Zhengzhou University, PR China. The respondents and their guardians provided their consents with written signatures. All respondents and their guardians are aware of all the personal information related in this study will be kept confidential and used only for the research purpose of this paper. Along with this, they are informed that no personal information or data will be shared with any third party.

## Author Contributions

ML and JL: conceptualization. MY, MO, and NH: methodology. ML and YX: software. NH, MO, and LA: validation and visualization. JL and YX: formal analysis. MY and MO: data curation. ML: writing—original draft preparation. JL and MO: writing—review and editing. JL and MY: supervision. MY: project administration. JL: funding acquisition. All authors have read and agreed to the published version of the manuscript.

## Conflict of Interest

The authors declare that the research was conducted in the absence of any commercial or financial relationships that could be construed as a potential conflict of interest.

## Publisher's Note

All claims expressed in this article are solely those of the authors and do not necessarily represent those of their affiliated organizations, or those of the publisher, the editors and the reviewers. Any product that may be evaluated in this article, or claim that may be made by its manufacturer, is not guaranteed or endorsed by the publisher.
